# National Comprehensive Cancer Network Guideline Recommendations of Cancer Drugs With Accelerated Approval

**DOI:** 10.1001/jamanetworkopen.2023.43285

**Published:** 2023-11-14

**Authors:** Edward R. Scheffer Cliff, Rachel S. Rome, Aaron S. Kesselheim, Benjamin N. Rome

**Affiliations:** 1 Program on Regulation, Therapeutics, and Law (PORTAL), Division of Pharmacoepidemiology and Pharmacoeconomics, Department of Medicine, Brigham and Women’s Hospital, Boston, Massachusetts; 2 Harvard Medical School, Boston, Massachusetts; 3 Palliative Medicine, Section of General Internal Medicine, Boston University School of Medicine and Boston Medical Center, Boston, Massachusetts

## Abstract

**Importance:**

Many cancer drugs are approved under the US Food and Drug Administration (FDA) accelerated approval pathway based on preliminary evidence. It is unclear how this limited evidence is integrated into the National Comprehensive Cancer Network (NCCN) guidelines, which are common references for clinicians and are used by public and private payers to determine reimbursement for oncology treatments.

**Objective:**

To analyze the NCCN guidelines’ assessments for cancer drug indications that received FDA accelerated approval compared with cancer drug indications that received FDA regular approval.

**Design, Setting, and Participants:**

This cross-sectional study analyzes FDA-approved indications for cancer drugs that were granted accelerated approval from program inception in 1992 to June 30, 2022. For each drug, the FDA-approved labeling was reviewed to identify all indications. All analyses were performed at the drug-indication level.

**Exposure:**

The exposure was FDA regulatory status as of October 2022, including regular approval, accelerated approval, accelerated approval converted to regular approval, and withdrawn accelerated approval.

**Main Outcomes and Measures:**

The level of evidence and consensus (category 1, 2A, 2B, and 3) and treatment preference (preferred, alternative preferred, other recommended, and useful in certain circumstances) ratings assigned by NCCN committees as of February 2023.

**Results:**

A total of 315 oncology indications for 100 drugs were analyzed. These indications included 156 (50%) with regular approval, 60 (38%) with accelerated approval, 78 (49%) with accelerated approval that was converted to regular approval, and 21 (13%) with withdrawn accelerated approvals. Among all indications, 105 (33%) were rated by the NCCN as having category 1 evidence, 185 (59%) with category 2A, 6 (2%) with category 2B, and 2 (1%) with category 3 evidence. Compared with indications with regular approval, those with accelerated approval were less frequently assigned category 1 evidence (47% vs 3%; *P* < .001) and were less often listed as preferred treatment options (58% vs 40%; *P* = .008). Among the 21 withdrawn accelerated approval indications, 8 (38%) remained in the NCCN guidelines, with most having level 2A evidence ratings.

**Conclusions and Relevance:**

This study found that cancer drug indications with accelerated approval were less likely to be assigned high-level evidence ratings and preferred status in the NCCN guidelines compared with indications with regular approval; most accelerated and regular approval drugs had low-quality evidence ratings but high levels of consensus among oncologists on NCCN committees. Greater clarity on the thresholds and definitions of evidence levels would make the NCCN guidelines more useful to clinicians, patients, and payers.

## Key Points

**Question:**

How are cancer drug indications with US Food and Drug Administration accelerated vs regular approval appraised in the National Comprehensive Cancer Network (NCCN) guidelines?

**Findings:**

This cross-sectional study of 315 cancer indications for 100 drugs found that indications with accelerated approval were assigned lower evidence and treatment preference ratings in the NCCN guidelines, compared with those with regular approval.

**Meaning:**

Findings in this study suggest that more robust evidence is needed to guide decision-making in oncology and that greater clarity regarding the evidence thresholds underlying NCCN classifications would make the guidelines more useful to clinicians, patients, and payers.

## Teaser

This cross-sectional study evaluates the basis for the supporting evidence and treatment preference ratings for cancer drugs and indications recommended by the National Comprehensive Cancer Network (NCCN) guidelines committees.

## Introduction

In the US, many new cancer drugs are approved via the accelerated approval pathway of the Food and Drug Administration (FDA).^1,2^ Under this pathway, certain drugs for serious conditions can be approved based on clinical trials that show improvements in surrogate measures, which are only reasonably likely to predict a clinical benefit.^2,3^ After the drugs are approved, the manufacturers are required to complete confirmatory trials to verify clinical benefit.^4^ However, drugs in the accelerated approval program have experienced substantial delays in drug manufacturers’ completion of confirmatory studies,^2,5^ have been priced at high levels despite uncertain clinical benefit,^6^ and have been slow to be withdrawn when confirmatory trials fail to demonstrate a clinical benefit.^4,7,8^

These regulatory challenges have been particularly pronounced among cancer drugs. For example, checkpoint inhibitors are now FDA-approved to treat more than 85 oncological indications,^9^ including dozens of accelerated approvals based on surrogate measures, such as response rate and progression-free survival.^10^ However, their efficacy and the strength of the correlation between surrogate measures and clinical end points^11,12^ vary substantially depending on cancer histological characteristics and subtypes,^9,10,13^ as acknowledged by the FDA^10^ and its Oncologic Drugs Advisory Committee.^14^

Although the accelerated approval status is included on drug labels, it is not clear whether this information factors in the treatment decisions of patients and oncologists. Furthermore, Medicare is required to cover all cancer drug indications that are approved by the FDA (including accelerated approval) that are listed in 1 of 5 compendia, perhaps the most prominent of which are the guidelines published by the National Comprehensive Cancer Network (NCCN).^15,16,17^ The NCCN guidelines combine evidence and expert opinion and are used by both public and private insurers to determine cancer drug coverage.^18^ In addition, the NCCN guidelines are widely used to inform oncologists’ clinical practice,^18,19^ and pharmaceutical companies frequently reference both a product’s inclusion in the guidelines and its category of evidence and preference in marketing materials and press releases.^20,21,22^

The NCCN guidelines frequently make recommendations on medications that extend beyond FDA-labeled indications; more than one-third of guideline recommendations relate to non–FDA-approved (off-label) uses.^18^ For some cancer drugs with accelerated approval, the NCCN guidelines continue to recommend their use even after confirmatory studies fail to demonstrate a clinical benefit or their indications are withdrawn by manufacturers.^7,23^

It is important to understand whether FDA regulatory status plays a role in the decisions and ratings by professional society guidelines, which in turn inform patients, oncologists, and payers. Given the centrality of the NCCN guidelines in cancer treatment and coverage determination, we undertook a cross-sectional study to analyze the NCCN guidelines’ assessments for cancer drug indications that received FDA accelerated approval compared with cancer drug indications that received FDA regular approval.

## Methods

In accordance with the Common Rule, this cross-sectional study was exempt from ethics review and informed consent because it was not considered human participant research. We followed the Strengthening the Reporting of Observational Studies in Epidemiology (STROBE) reporting guideline.

### Cohort

We identified cancer drugs that were granted accelerated approval from the program’s inception in 1992 through June 30, 2022, using a public FDA list.^24^ For each drug, we reviewed the FDA approved labeling as of October 19, 2022, to identify all indications, including indications that were granted regular approval and accelerated approval. Indications with no NCCN guidelines (eg, naxitamab for neuroblastoma) and those that were tumor-type agnostic (eg, dabrafenib plus trametinib for solid tumors with *BRAF* V600E alteration) were excluded. We also excluded indications for gemtuzumab ozogamicin because the drug was withdrawn then reapproved with a different dosing strategy and a different indication. All analyses were performed at the drug-indication level.

### Exposure and Outcome

The exposure variable for this study was FDA regulatory status as of October 19, 2022. Exposure included indications that were initially granted regular approval, accelerated approval indications that were converted to regular approval after confirmatory studies, accelerated approval indications that were withdrawn by the manufacturer (typically after failed confirmatory studies), and indications that remained under accelerated approval.^7^

The relevant NCCN Clinical Practice Guidelines in Oncology^25^ for each indication were reviewed as of February 10, 2023. These guidelines are updated frequently, and all guidelines reviewed in this study had been updated as of April 4, 2022.

Each drug-indication combination was assessed for its level of evidence rating and level of treatment preference rating given by the relevant NCCN committee. These committees, typically comprising 20 to 30 subspecialty experts, meet multiple times per year to appraise and discuss the most recent evidence to reach a consensus on practical recommendations for clinicians. The committees assign ratings to treatment approaches that reflect the level of evidence supporting the treatment’s use, as well as preference ratings that compare the treatment’s efficacy with that of other available treatment options (Table 1).^26^

**Table 1.** National Comprehensive Cancer Network (NCCN) Categories of Level of Evidence and Consensus and Treatment Preference Ratings

NCCN ratings	NCCN definition
Level of evidence and consensus	
1	High level evidence with uniform panel consensus (>85% of votes)
2A	Lower level evidence with uniform panel consensus (>85% of votes)
2B	Lower level evidence without uniform consensus (50%-85% of votes)
3	Any level evidence but with major disagreement (25%-50% of votes)
Treatment preference	
Preferred	Based on superior efficacy, safety, and evidence and, when appropriate, affordability
Alternative preferred	No definition given
Other recommended	May be somewhat less efficacious, more toxic, or based on less mature data; or significantly less affordable for similar outcomes
Useful in certain circumstances	May be used for select patient populations defined with recommendation

Evidence ratings were category 1 (high level of evidence with uniform consensus), category 2A (lower level of evidence with uniform consensus), category 2B (lower level of evidence without uniform consensus but with no major disagreement), and category 3 (any level of evidence but with major disagreement). We separately defined when drug indications were removed or excluded from the guidelines. Categories of treatment preference ratings included preferred, alternative preferred, other recommended, or useful in certain circumstances.

### Statistical Analysis

We descriptively analyzed level of evidence and treatment preference ratings for cancer indications, stratified by approval status. We used Fisher exact test, with 2-sided hypothesis tests and a significance level of *P* < .05 established a priori, to compare evidence and preference ratings of indications with accelerated approval against those with converted accelerated and regular approvals. Fisher exact test was chosen to analyze these nominal variables due to a small sample size. Analyses were performed in Microsoft Excel for Mac, version 16.70 (Microsoft Corp), and R, version 4.3.0 (R Foundation for Statistical Computing).

## Results

We included 315 cancer indications for 100 drugs. Overall, 156 indications (50%) were initially granted regular approval and 159 (50%) were initially granted accelerated approval. Among indications with accelerated approval, 78 (49% of accelerated approvals) were converted to regular approval, 21 (13%) were withdrawn, and 60 (38%) remained as accelerated approvals as of October 2022 (Table 2). Of the 100 drugs, 45 (45%) had indications with only accelerated approval, whereas 55 (55%) had indications with both accelerated and regular approvals. Among all indications, the most common cancer types were non–small cell lung cancer (45 [14%]), breast cancer (27 [9%]), B-cell lymphomas (21 [7%]), colorectal cancer (18 [6%]), and multiple myeloma (18 [6%]) (Table 2). A plurality of 72 indications (23%) were for 1 of 6 checkpoint inhibitor drugs (programmed cell death 1 inhibitor or programmed cell death ligand 1 inhibitors). Thirteen indications (4%) were approved in 1992 to 2000, 75 (24%) in 2001 to 2010, 189 (60%) in 2001 to 2010, and 38 (12%) in 2021 to 2022.

**Table 2.** Characteristics of Cancer Indications by Cancer Subtype and FDA Approval Status

Cancer subtype	FDA approval status, No. (%)^a^				
	Regular approval (n = 156)	Current AA (n = 60)	Converted AA (n = 78)	Withdrawn AA (n = 21)	Total (N = 315)
Acute leukemia	6 (4)	2 (3)	3 (4)	1 (5)	12 (4)
B-cell (non-Hodgkin) lymphoma	2 (1)	12 (20)	2 (3)	3 (24)	21 (7)
Bladder cancer	4 (3)	3 (5)	3 (4)	3 (14)	13 (4)
Breast cancer	14 (9)	1 (2)	10 (13)	2 (10)	27 (9)
Chronic lymphocytic leukemia	4 (3)	0	4 (5)	2 (10)	10 (3)
Chronic myeloid leukemia	4 (3)	1 (2)	10 (13)	0	15 (5)
Colorectal cancer	10 (6)	3 (5)	5 (6)	0	18 (6)
Gastric and/or esophageal cancer	10 (6)	1 (2)	0	1 (5)	12 (4)
Head and neck cancer	7 (4)	0	1 (1)	0	8 (3)
Hepatocellular carcinoma	4 (3)	3 (5)	0	1 (5)	8 (3)
Melanoma	8 (5)	0	5 (6)	0	13 (4)
Multiple myeloma	9 (6)	2 (3)	6 (8)	1 (5)	18 (6)
NSCLC	25 (16)	8 (13)	11 (14)	1 (5)	45 (14)
Ovarian cancer	4 (3)	0	4 (5)	0	8 (3)
Kidney cancer	12 (7)	0	2 (4)	0	14 (4)
Thyroid cancer	4 (3)	4 (7)	0	0	8 (3)
Other	29 (19)	20 (33)	12 (15)	4 (19)	65 (14)

Abbreviations: AA, accelerated approval; FDA, US Food and Drug Administration; NSCLC, non–small cell lung cancer.

^a^ Percentage represents the column percentage.

Among all 315 indications, 105 (33%) were supported by NCCN category 1 evidence ratings, 185 (59%) by category 2A, 6 (2%) by category 2B, and 2 (1%) by category 3; 17 indications (7%) were removed or excluded from the guidelines. For the NCCN category of treatment preference ratings, 153 indications (49%) were preferred, 5 (2%) were alternative preferred, 55 (17%) were other recommended, and 38 (12%) were useful in certain circumstances; 47 drug-indication combinations (15%) had no category of preference listed.

**Figure 1.**
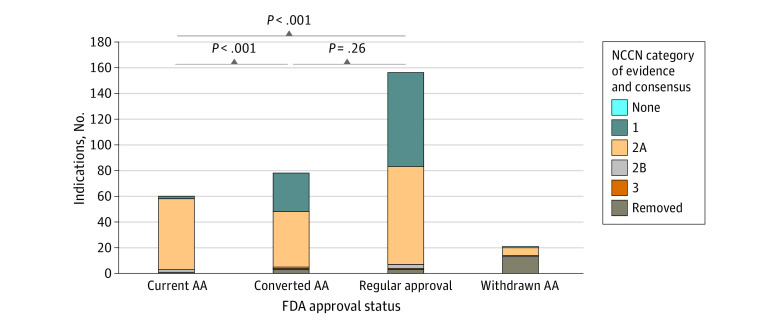
National Comprehensive Cancer Network (NCCN) Category of Evidence Ratings Stratified by US Food and Drug Administration (FDA) Approval Status AA indicates accelerated approval.

**Figure 2.**
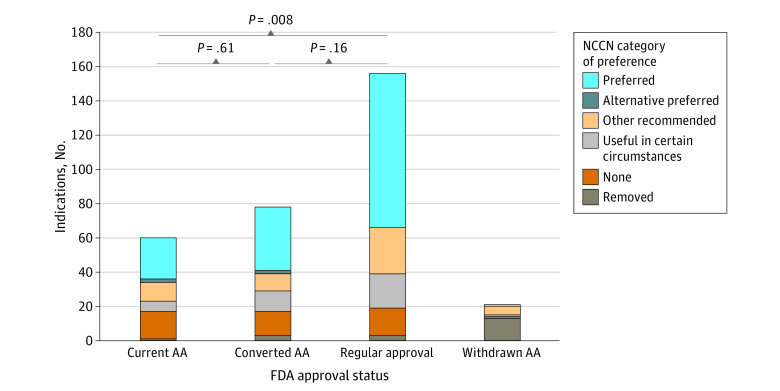
National Comprehensive Cancer Network (NCCN) Category of Preference Ratings Stratified by US Food and Drug Administration (FDA) Approval Status AA indicates accelerated approval.

Compared with indications that were initially granted regular approval, indications with current accelerated approval less frequently received category 1 evidence ratings (47% vs 3%; *P* < .001) (Figure 1) and were less often categorized as preferred (58% vs 40%; *P* = .008) (Figure 2). Compared with indications with current accelerated approval, those converted to regular approval had higher evidence ratings (3% vs 38% category 1; *P* < .001) and were similarly likely to be preferred (40% vs 47%; *P* = .61). Converted indications were also similarly likely to have category 1 evidence vs those with regular approval (38% vs 47%; *P* = .26) and similarly likely to be preferred (47% vs 58%; *P* = .16)

Among the 21 withdrawn accelerated approval indications, 8 (38%) remained in the NCCN guidelines, 6 of which had category 2A evidence ratings (Table 3). One of these indications was listed as preferred (romidepsin for T-cell lymphoma). In all 8 cases, the guidelines were updated after the withdrawal, and these recommendations remained.

**Table 3.** Withdrawn Accelerated Approval Indications Remaining in the NCCN Guidelines

Drug	Indication	NCCN category of evidence ratings	NCCN category of treatment preference ratings	Date of withdrawal	Date of guideline update
Fludarabine	Chronic lymphocytic leukemia	2A	Useful in certain circumstances	Dec 31, 2011	Jan 25, 2023
Idelalisib	Small lymphocytic lymphoma	2A	Other recommended	Feb 18, 2022	Jan 25, 2023
Romidepsin	T-cell lymphoma	2A	Preferred	Jul 30, 2021	Jan 5, 2023
Pembrolizumab	SCLC	2A	Other recommended	Mar 30, 2021	Dec 21, 2022
Nivolumab	SCLC	2A	Other recommended	Dec 29, 2020	Dec 21, 2022
Nivolumab	Hepatocellular carcinoma	2A	Other recommended	Jul 23, 2021	Oct 14, 2022
Atezolizumab	Urothelial carcinoma, platinum-ineligible	3	Useful in certain circumstances	Dec 2, 2022	Feb 9, 2023
	Urothelial carcinoma, PD-L1 positive^a^	2B	Other recommended		

Abbreviations: NCCN, National Comprehensive Cancer Network; PD-L1, programmed cell death ligand 1; SCLC, small cell lung cancer.

^a^ US Food and Drug Administration definition: PD-L1–stained tumor-infiltrating immune cells of any intensity covering 1% or greater of the tumor area. NCCN definition: PD-L1–stained tumor-infiltrating immune cells covering 5% or greater of the tumor area.

## Discussion

In this cross-sectional study of 315 cancer drug indications, those with accelerated approval were less likely to receive the highest evidence rating and preferred status in the NCCN guidelines compared with those that were granted regular approval. However, most indications, including those with both accelerated and regular approvals, had uniform expert endorsement despite low-quality evidence (ie, category 2A). Additionally, over one-third of accelerated approval indications that had been withdrawn by the FDA continued to be recommended in the guidelines at a level representing uniform consensus.

The NCCN guidelines often identified indications with accelerated approval as having lower-level evidence than indications with regular approval, a finding consistent with that of prior studies.^2,3,27^ Indications that were granted accelerated approval were also less likely to be rated as preferred treatments than indications with regular approval. This finding is problematic because the FDA’s accelerated approval program is specifically designed to expedite approval of drugs that fulfil unmet medical needs; the guideline ratings suggest that, often, other regimens are preferred over those with accelerated approval, which raises questions about whether the basic regulatory criteria for accelerated approval were met.

Although the lower evidence standard for drugs with accelerated approval is well recognized, we also found that regular approvals were frequently supported by lower-level evidence: only 47% of indications that were initially granted regular approval and 38% of accelerated approvals that were converted to regular approval were rated as having category 1 evidence. Previous studies have demonstrated that the FDA has increasingly relied on nonrandomized trials and studies using surrogate measures of efficacy as trial end points, rather than clinical outcomes, for confirmatory studies of drugs with accelerated approval and for drugs with regular approvals.^7,10^ This finding is problematic due to the frequency with which drugs with promising efficacy in phase 2 studies fail to show benefit in phase 3 randomized clinical trials,^28^ especially in oncology.^29^

Better clinical evidence is needed to guide the optimal use of many cancer drugs and ensure they offer clinically meaningful benefits for patients. The large number of drugs with category 2A evidence raises concerns about the clarity and usefulness of this rating to practicing clinicians. For example, in B-cell lymphoma, selinexor, a novel drug tested in 1 single-arm phase 2 trial of 134 patients, is labeled category 2A evidence, and ibrutinib, which has been tested in thousands of patients across multiple randomized trials, is also labeled category 2A. Among the NCCN’s definitions for its thresholds for consensus,^26^ only 85% of votes are required to be classified as uniform consensus, and the NCCN does not specify thresholds for its delineation between high-level and lower-level evidence. Clearer definitions or thresholds for high-level and lower-level evidence, potentially with the addition of an intermediate category of evidence, may also make the recommendations more informative. Although the NCCN committees include academic oncologists from a broad range of institutions, these levels of consensus may be undermined by the widespread financial conflicts of interest among committee members^30,31^; thus, it is possible that, in some cases, the NCCN guidelines represent optimistic interpretations of evidence supporting novel drugs.

A substantial proportion of indications with accelerated approval that were withdrawn by the drug’s manufacturer continued to be recommended in updated NCCN guidelines, emphasizing that these guidelines were frequently broader than FDA-approved labeling.^18,32^ Because these drugs remained marketed for other indications, oncologists were typically free to continue using them off-label for the withdrawn indications. Furthermore, because these indications remained in the NCCN guidelines, Medicare was obligated to continue providing coverage despite a lack of demonstrated effectiveness, and many states also have laws requiring coverage by insurers in the private market.^33^

The results of this study have several implications for policymakers. In February 2023, the Center for Medicare and Medicaid Innovation proposed a new model to lower reimbursement for drugs that were approved via the accelerated approval pathway, which would also incentivize manufacturers to complete confirmatory trials of their drugs in a timely fashion.^34^ A similar idea has been proposed for Medicaid.^35^ The results suggest that policymakers may need to think more broadly about how to align reimbursement with level of evidence because high-quality evidence was lacking even for many drugs with regular approval. Furthermore, although the FDA has recently become more diligent about working with manufacturers on withdrawing indications from drugs with accelerated approval that did not demonstrate benefit in confirmatory trials,^7^ these withdrawn indications frequently continue to be recommended in the NCCN guidelines, suggesting continued reimbursement by Medicare. The Centers for Medicare and Medicaid Services could rectify this issue by explicitly allowing coverage exclusions for withdrawn indications.

### Limitations

This study has several limitations. The cohort included only drugs with at least 1 accelerated approval indication. The findings may not be generalizable to cancer drugs that have only indications with regular approval. There was also heterogeneity in the guidelines by cancer type; for example, not all guidelines listed categories of preference. Evaluation of the NCCN Evidence Blocks, which are simplified visual tools to help inform clinicians about the evidence supporting particular drugs, was outside the scope of this study.

## Conclusions

This cross-sectional study found that cancer drug indications with accelerated approval had lower evidence and treatment preference ratings in the NCCN guidelines than indications with regular approval from the FDA; however, even among drugs with regular approval, most indications were supported by lower-level evidence. Greater clarity regarding the NCCN thresholds and definitions of level of evidence may make the NCCN guidelines more useful to clinicians, patients, and payers.

## Notes

Supplement.
Data Sharing Statement
