# Supplementary material for: National Comprehensive Cancer Network Guideline Recommendations of Cancer Drugs With Accelerated Approval
Source: JAMA Netw Open. 2023 Nov 14;6(11):e2343285. doi: 10.1001/jamanetworkopen.2023.43285 (PMC10646727; doi:10.1001/jamanetworkopen.2023.43285)
Supplement: Supplement. — Data Sharing Statement [file jamanetwopen-e2343285-s001.pdf]

Data Sharing Statement

Cliff. National Comprehensive Cancer Network Guideline Recommendations of Cancer Drugs With Accelerated Approval. *JAMA Netw Open*. Published November 14, 2023. doi:10.1001/jamanetworkopen.2023.43285

Data
Data available: Yes
Data types: Data (not involving human participants)
How to access data: https://www.nccn.org/
https://www.accessdata.fda.gov/scripts/cder/daf/index.cfm
When available: With publication

Supporting Documents
Document types: None

Additional Information
Who can access the data: anyone making reasonable request of data
Types of analyses: for any reasonable purpose
Mechanisms of data availability: with investigator support
